# Artificial Intelligence improves follow-up appointment uptake for diabetic retinal assessment: a systematic review and meta-analysis

**DOI:** 10.1038/s41433-025-03849-4

**Published:** 2025-05-30

**Authors:** Masoud Rahmati, Lee Smith, Mapa Prabhath Piyasena, Michael Bowen, Laurent Boyer, Guillaume Fond, Abdolreza Kazemi, Dong Keon Yon, Hayeon Lee, Tarnjit Sehmbi, Sanjiv Ahluwalia, Shahina Pardhan

**Affiliations:** 1 CEReSS-Health Service Research and Quality of Life Center, Assistance Publique-Hopitaux de Marseille, Aix-Marseille University, Marseille, France; 2 CRSMP, Center for Mental Health and Psychiatry Research – PACA, Marseille, France; 3 Department of Physical Education and Sport Sciences, Faculty of Literature and Human Sciences, Lorestan University, Khoramabad, Iran; 4 Department of Physical Education and Sport Sciences, Faculty of Literature and Humanities, Vali-E-Asr University of Rafsanjan, Rafsanjan, Iran; 5 Centre for Health, Performance, and Wellbeing, Anglia Ruskin University, Cambridge, UK; 6 Vision and Eye Research Institute, School of Medicine, Anglia Ruskin University, Young Street, Cambridge, UK; 7 The College of Optometrists, London, UK; 8 Center for Digital Health, Medical Science Research Institute, Kyung Hee University Medical Center, Kyung Hee University College of Medicine, Seoul, Republic of Korea; 9 Department of Pediatrics, Kyung Hee University College of Medicine, Seoul, Republic of Korea; 10 Head of School of Medicine, Anglia Ruskin University, Chelmsford, England; 11 Centre for Inclusive Community Eye Health, Anglia Ruskin University, Cambridge, UK

**Keywords:** Health care, Scientific community

## Abstract

**Background/Objectives:**

Artificial intelligence (AI) assessment of diabetic retinopathy (DR) instead of scarce trained specialists could potentially increases the efficiency and accessibility of screening programs. This systematic review aims to systematically examine the uptake of follow-up appointments with initial computer-based AI and human graders of DR.

**Methods:**

We conducted a systematic review and meta-analysis by screening articles in any languages in PubMed, MEDLINE (Ovid), EMBASE, Web of Science, Cochrane CENTRAL and CDSR published from database inception up to 20^th^ August 2024. We used random-effects meta-analysis to pool the results as odds ratios (OR) with corresponding 95% confidence intervals (CI).

**Results:**

Data from a total of 20,108 patients with diabetes (6476 participants graded using AI and 13,632 participants graded by human-graders; age range of the participants 5 to 67 years) from six studies were included. The result of the pooled meta-analysis showed that initial AI assessment of DR significantly increased uptake of follow-up appointments compared to human grader-based (OR = 1.89, 95% CI 1.78–2.01, *P *= 0.00001).

**Conclusions:**

The present systematic review and meta-analysis suggest that initial AI-based algorithm for screening DR is associated with an increased uptake of follow-up examination. This is most likely due to instant results being made available with AI based algorithms when compared to a delay in assessment with human graders.

## Introduction

The number of people with diabetes is increasing rapidly globally, with more than 529 million people living with diabetes worldwide in 2021. This figure is estimated to increase to 1.3 billion by 2050 [1]. Diabetic retinopathy (DR), a common micro-vascular complication of diabetes, is a leading cause of vision impairment and blindness. DR is a major global public health concern and represents a substantial burden to health systems globally [2]. Although early diagnosis and treatment of diabetic eye disease and complications through DR screening reduces vision loss by 98% [3], our review team members have recently shown that the global DR screening adherence rate was 66.9% in high-income countries and 39.3% in low-and-middle-income countries [4]. Furthermore, despite the implementation of DR screening in multiple countries, the rates at which those identified with referrable DR take up those referrals remains lower than desired [5].

Over the last two decades, poor DR screening adherence and follow-up referral service uptake has been addressed partially by the introduction of telemedicine [6, 7]. Recent advances in computer-based analysis using artificial intelligence (AI) systems for diagnosing diabetic eye diseases has started the next chapter of DR screening, with machine learning and deep learning techniques being employed to analyse medical images, predict disease outcomes, and assist in clinical decision-making [8–13]. AI systems are capable of detecting subtle retinal changes from fundus images with high accuracy, offering a scalable and cost-effective solution to the growing global burden of DR [8–13].

Assessment of DR screening images by AI, instead of by scarce trained specialists could potentially increases the efficiency and accessibility of screening programs, and may even decrease cost of screening [14]. Current evidence suggests that AI algorithms have acceptable performance in DR screening for using fundus images compared to human graders [15, 16].

While previous research has examined the impact of AI assessment of DR on uptake of follow-up of referral service in various settings [8–13], to date, there is no meta-analysis to summarize the pool effects of the available evidence. An improved understanding of the association between AI assessment of DR and uptake of follow up of appointments after identifying a referrable level of DR is needed to inform public policy, public health programme planning, and allocation of limited healthcare resources. We conducted a systematic review and meta-analysis to summarize the up-to-date evidence to examine any association between AI assessment of DR and uptake of further follow-up hospital eye service appointments.

## Methods

The present systematic review and meta-analysis followed the methodological guidelines from the Cochrane Handbook for Systematic Reviews [17] and the PRISMA (Preferred Reporting Items for Systematic Review and Meta-Analyses) statement 2020 to conduct and report the association between AI assessment of DR and adherence to routine digital screening and follow-up hospital eye services uptake of DR screening (Table S1) [18]. The systematic review was pre-registered with the International Prospective Register of Systematic Reviews (PROSPERO registration No: CRD42024590282).

### Search strategy

Two researchers (MR and DKY) electronically searched five databases, including PubMed, MEDLINE (Ovid), EMBASE, Web of Science, and Cochrane CENTRAL and CDSR from database inception up to 20^th^ August 2024. Disagreements were resolved through discussion with a third reviewer (SP). The search strategy and terms related to AI assessment and follow-up service uptake of DR screening are provided in Supplementary Table 2. To find all eligible articles, we searched all reference lists of included studies related to AI assessment and follow-up service uptake of DR screening, and no language restrictions for studies with an English summary were applied.

### Eligibility criteria

The present systematic review and meta-analysis adhered to the PICOTS criteria for inclusion of studies [19]. PICOTS: Participants include patients with diabetes who were assessed with AI for DR screening; Intervention group were participants who had initial AI-based assessment of DR; Comparison includes control group of people with diabetes who were assessed with human-based DR screening; Outcome includes studies reporting rate of adherence (or uptake) for follow up appointments to assess DR; Time includes follow-up of appointments; and Setting includes DR screening using digital retinal imaging. We included both cohorts, and randomized-control trial (RCT) studies that evaluated AI assessment of DR and follow-up appointment uptake after identifying a referrable level of DR. For clarification, “follow-up appointments for DR” refers to any subsequent visits scheduled for individuals either for routine retinal screening or at a healthcare provider if referral retinopathy was seen. We have then further elaborated on this term to clarify whether the follow up was a “follow up routine digital screening,” referring to the regularly scheduled retinal screenings aimed at detecting signs of diabetic retinopathy in individuals with diabetes, and a “follow-up hospital eye service” referring to specialized follow-up care provided by ophthalmology departments in hospitals for patients diagnosed with referrable level DR or those at high risk of developing sight threatening DR. In our study, moderate non-proliferative DR (NPDR) and above (including macular oedema) were considered as conditions that warranted the uptake of follow-up hospital eye services for DR screening. We excluded studies that lacked data to be able to calculate odds ratio (OR), or association between AI assessment and follow-up service uptake of DR screening. Studies were excluded if their primary research question was not exploring AI assessment and routine digital screening or follow-up service uptake of DR screening. We also excluded studies without a control group of human-based DR workflow for DR screening. Further, studies were excluded if they were narrative literature reviews (although their reference lists were explored for potentially eligible studies). Two reviewers (MR and DKY) independently selected the articles at title and abstract screening stage and full-text report screening stage, and disagreements were resolved through discussion with a third reviewer (SP).

### Data extraction and quality assessment

Data were extracted using Covidence systematic review software (version 2, Veritas Health Innovation, Melbourne, VIC, Australia) on a spreadsheet pre-designed following Cochrane guidelines. The following data were extracted from the eligible studies: author and year, study design, country, age of participants, sample size, patient characteristics, digital retinal imaging device used, setting of DR screening, type of AI algorithm and interpretation, rate of adherence to routine digital screening, follow-up period and rate of uptake at hospital eye services. The primary outcome was to estimate the association between AI assessment of DR and adherence to routine digital screening and follow-up hospital eye service uptake after identifying a referrable level of DR. The secondary outcomes were to explore any associations between DR screening adherence by different age category and level of service delivery in DR screening care pathway. The quality of included studies were assessed using the Newcastle–Ottawa Scale (NOS) [20] and Cochrane risk of bias tool (RoB 2) according to the “Cochrane Handbook for Systematic Reviews of Interventions” [21] for cohort and RCT studies, respectively. Data extraction and quality assessment were independently performed by two reviewers (MR and DKY), and through discussion with a third reviewer (SP) in case of any disagreements before conducting meta-analysis.

### Statistical analyses

Outcomes were pooled and expressed as ORs with corresponding 95% confidence intervals (CI) based on a one-stage approach and the random-effects estimated using the inverse variance method [22, 23]. We used the Peto odds ratio method to estimate the odds of follow-up appointments for DR. This method has been shown to be beneficial due to its higher accuracy when addressing rare events and small intervention effects, which was a characteristic of certain studies included in our analysis. Additionally, we applied the Mantel-Haenszel method to combine the results of different studies and to obtain a pooled odds ratio. This approach was helpful in controlling for potential confounders, allowing us to provide a more robust estimate of the effect of AI on the uptake of follow-up appointments and hospital eye services for DR [24–26]. The degree of between-study heterogeneity of included studies was explored using Cochran’s Q statistics and I-squared (*I*^*2*^; low: 0–40%, moderate: 30–60%, substantial: 50–90%, and considerable: >75%) to estimate heterogeneity [4, 26–28]. We also performed a subgroup analyses based on different age categories in included studies (5–21 years versus ≥ 18 years) and by level of DR service delivery (routine digital screening and follow-up hospital eye service uptake). Further, to assess the robustness of summary estimates and to detect a large proportion of heterogeneity of any particular study, sensitivity analysis was performed by the leave-one-out method. All meta-analyses in the current study were conducted using Review Manager (version 5.4; The Nordic Cochrane Centre, Copenhagen, Denmark), and a two-sided P value less than 0.05 was considered statistically significant.

## Results

**Fig. 1:**
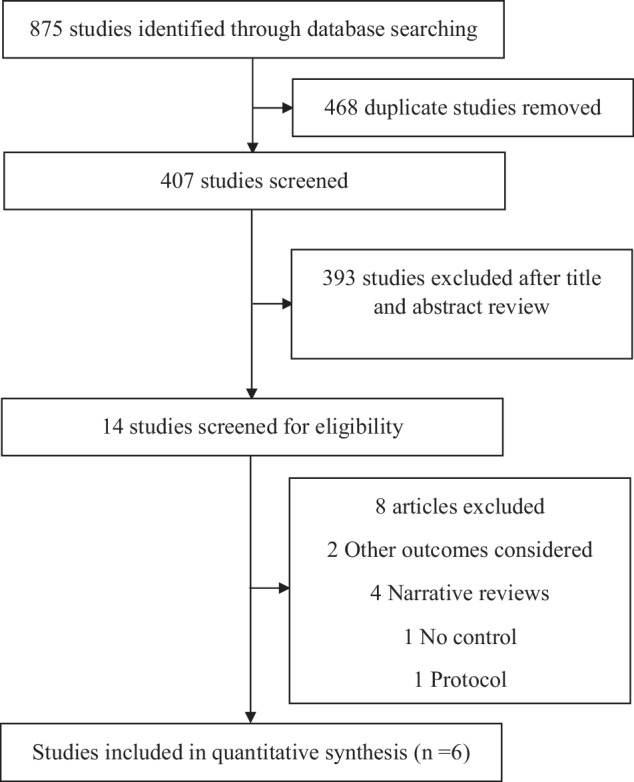
PRISMA flow diagram of study selection.

Starting from 468 records after duplicate removal, we excluded 393 studies at the title and abstract screening stage, and a further eight following full-text review, resulting in six publications being included. All excluded studies after full-text assessment, with reason for exclusion, are listed in Supplementary Table 3, and the article selection flow diagram is represented in Fig. 1. The details of the characteristics of the included studies are presented in Table 1. The eligible studies were published between 2021 and 2024.

**Table 1:** General characteristics of included studies.

Study	Country	Study design (n)	Setting	AI integration	Sample Size	Mean age (y)	Patient characteristic	Devise used	Imaging Strategy	AI interpretation	Follow-up period	Follow-up rate
Dow, [8]	USA	Cohort	Primary care and endocrinology clinics	AI as an assistance for human graders (STATUS)	AI: 279 Con: 117	55	Patients with diabetes mellitus without a prior DR diagnosis or a DR exam in the past 12 months	Topcon NW400 camera	Non-mydriatic fundus; 2-field (optic disc and center-cantered)	IDx-DR through a secure cloud-based algorithm	Within 3 months after screening	AI: 35.5% (99/279) Con: 12% (14/117)
Huang, [9]	USA	Cohort	Primary care	Fully automated AI (LumineticsCore®)	2019: AI: 5505 Con: 12169 2021: AI: 5580 Con: 12010	≥ 18	Patients with diabetes mellitus who were managed at primary care sites of Johns Hopkins Medicine in the calendar years 2019 and 2021	NR	NR	IDx-DR through a secure cloud-based algorithm	Within 12 months after screening	AI: 54.5% (3041/5580) Con: 40.3% (4840/12010)
Liu, [10]	USA	Cohort	Primary care	AI as an assistance for human graders (EyeArt 2.0)	AI: 92 Con: 974	≥ 18	Patients with diabetes mellitus without a prior DR diagnosis or a DR exam in the past 12 months	CR-2 retinal camera	Non-mydriatic fundus	EyeArt 2.0 automated DR screening software	Within 3–12 months after screening	AI: 55.4% (51/92) Con: 18.7% (182/974)
Mathenge, [11]	Rwanda	RCT	Diabetes clinics	AI as an assistance for human graders	AI: 136 Con: 139	≥ 18	Patients with diabetes mellitus without a prior DR diagnosis or a DR exam in the past 12 months	Topcon NW400 camera	Non-mydriatic and mydriatic if image quality was poor; 2-field (optic disc and center-cantered)	Inception ResNet version 2 convolutional neural network architecture	Within 1 months after screening	AI: 51.5% (70/136) Con: 39.6% (55/139)
Wolf, [13]	USA	Cohort	Multi-disciplinary paediatric diabetic clinic	Fully automated AI (LumineticsCore®)	310	5–21	Youth with T1D aged ≥10 years who met the criteria for diabetic eye disease screening per American Diabetes Association 2021 guidelines without a prior DR diagnosis or a DR exam in the past 6 months	Topcon NW400 camera	Non-mydriatic fundus	IDx-DR through a secure cloud-based algorithm	NR	AI: 95% (287/308) Before AI implementation: 49% (152/310)
Wolf, [12]	USA	RCT	Academic paediatric diabetes center	Fully automated AI (LumineticsCore®)	AI: 81 Con: 82	5–21	Youth with T1D aged ≥10 years who met the criteria for diabetic eye disease screening per American Diabetes Association 2021 guidelines without a prior DR diagnosis or a DR exam in the past 6 months	Topcon NW400 camera	Non-mydriatic fundus	IDx-DR through a secure cloud-based algorithm	Within 6 months after screening	Adherence: AI: 100% (81/81) Con: 22% (18/82) Follow-up: AI: 64% (16/25) Con: 22% (18/82)

*RCT* randomized-control trial, *STATUS* Stanford Teleophthalmology Automated Testing and Universal Screening; LumineticsCore®, formerly known as IDx-DR, was applied in primary care settings that were part of the Johns Hopkins Medicine system EyeArt 2.0, was applied in a primary care setting at the Primary Care Medicine Clinic of Barnes Jewish Hospital in Saint Louis, MO; AI, Artificial Intelligence; Con, Control.

A total of 20,108 patients with diabetes were included in this analysis (6476 participants graded using AI and 13,632 participants graded by human graders). Included studies were from two countries (five in USA and one in Rwanda). The age of the participants ranged from 5 to 67 years with two studies on children and young adults [12, 13] (5–21 years). Rate of follow-up uptake after the initial screening varied from 35.5% to 100% in AI-assessed patients and 18.7% to 40.3% in control patients (assessed by human graders). In four studies, AI interoperation was done by IDx-DR through a secure cloud-based algorithm [8, 9, 12, 13], in one study by EyeArt 2.0 automated DR screening software [10], and in one other study by Inception ResNet version 2 convolutional neural network architecture [11]. Participants of included studies were screened using non-mydriatic fundus photographs taken with either Topcon nw400 camera (Welch Allyn Inc., Skaneateles Falls, NY) [8, 11–13] or CR-2 retinal camera (Canon U.S.A, Inc., Melville, NY) [10]. In all included studies, patients without a prior DR diagnosis were included.

### Risk of bias of included studies

The randomization process was described in all studies included and was appropriate for two RCT studies and in no study were allocations concealed. In addition, the reported rate of drop out in one study was high (60.4%), hence their attrition bias was considered high [11]. By checking the available protocols of included studies, the selective reporting bias in both included RCTs was judged as low risk. No risk of other biases was identified in both included RCTs. Further, all included cohort studies were of medium to high quality, with NOS scores of between 8 and 9 (Supplementary Table 4).

### Association between artificial intelligence assessment of DR and adherence to routine digital screening and follow-up hospital eye service uptake of DR

**Fig. 2:**
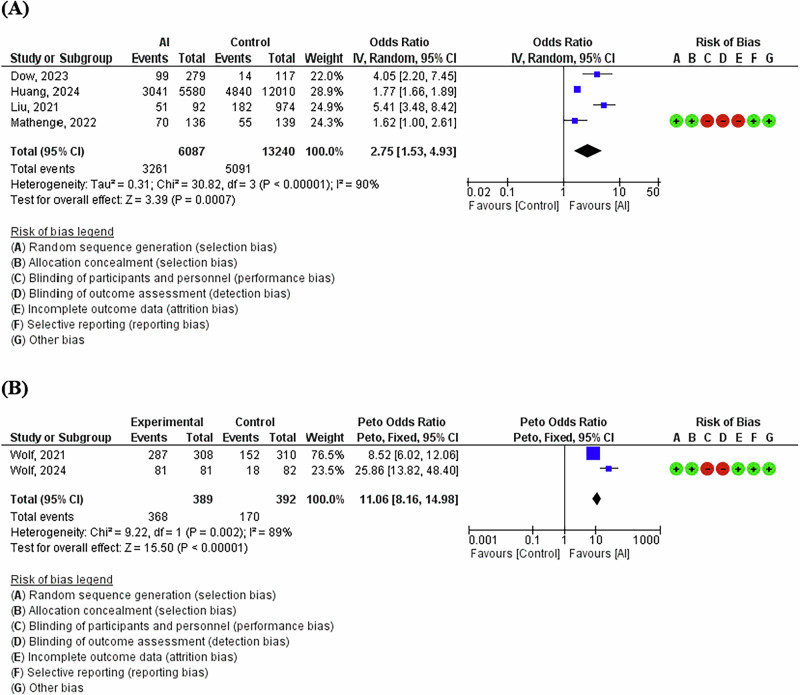
Association Between AI-Based Assessment and Follow-Up Uptake for Diabetic Retinopathy. **A** Forest plot of the association between artificial intelligence assessment uptake of follow-up appointments for DR in adults. **B** Forest plot of the association between artificial intelligence assessment uptake of follow-up appointments for DR in children.

Six studies involving 20,108 people with diabetes (6476 participants graded using AI and 13,632 participants graded by human graders) reported uptake of follow-up examination after AI and human grader assessment of DR [8–13]. Pooled analyses including studies on adult participants (>18 years, four studies, 3261 participants graded using AI and 5091 participants graded by human graders) [8–11] showed OR = 2.75 for the association between AI assessment uptake of follow-up appointments for DR (95% CI 1.53–4.93, *P *= 0.0007) (Fig. 2A). Analysing the two studies on youths (5–21 years old) separately (368 participants graded using AI and 170 participants graded by human graders) [12, 13] showed a much higher OR = 11.06 (95% CI 8.16–1498, *P *= 0.00001) (Fig. 2B).

Pooled meta-analysis of both adult and youth participants found that AI assessment significantly increased the uptake of follow-up appointments for DR compared to human graders (OR = 1.89, 95% CI 1.78–2.01, *P *= 0.0001) (Supplementary Fig. 1).

**Fig. 3:**
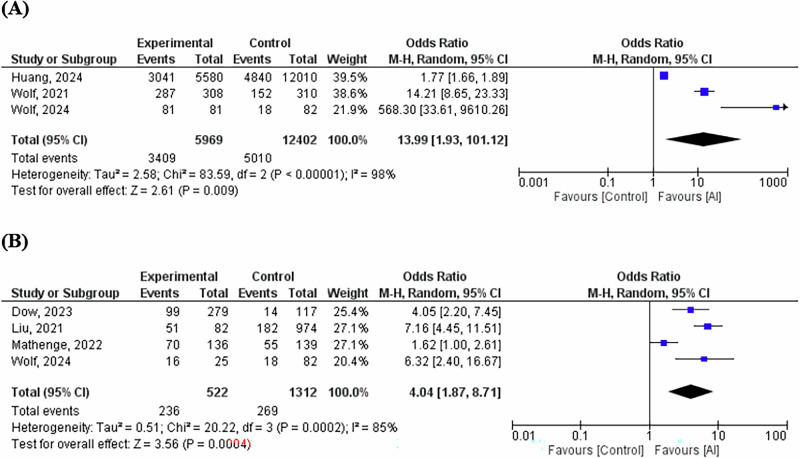
Association Between AI-Based Assessment of Diabetic Retinopathy and Screening or Follow-Up Adherence. **A** Forest plot of the association between artificial intelligence assessment of DR and adherence to routine digital screening. **B** Forest plot of the association between artificial intelligence assessment of DR and follow-up hospital eye service uptake.

Moreover, we found OR = 13.99 (95% CI 1.93–101.12, *P *= 0.009) in meta-analysis of three studies [9, 12, 13] evaluated the association between AI assessment of DR and adherence to routine digital screening (Fig. 3A). Further, OR = 4.04 (95% CI 1.87–8.71, *P *= 0.0002) was found in meta-analysis of four [8, 10–12] studies evaluated the association between AI assessment and follow-up hospital eye service-uptake of DR screening (Fig. 3B).

## Discussion

The Lancet Commission on Eye Health highlighted the importance of eye health in contributing towards the achievement of the Sustainable Development Goals [29]. With the increasing number of people with diabetes and DR, urgent attention is needed in developing innovative screening strategies for conditions like DR.

The results of our systematic review and meta-analysis showed significantly higher likelihood of follow-up appointment uptake with AI, compared to conventional human grading. This was markedly high for youth (OR 11.06; age cutoff at 21 years) compared to the adults (OR 2.75). To our knowledge, the present systematic review and meta-analysis is the first evidence synthesis on this topic. DR is a major cause of blindness among the economically active working age population [30]. Despite concerted effort in many countries, with varied income settings, DR is predicted to be a major cause of sight loss and is a significant public health challenge with the rapid rise of prevalence of diabetes and increased life expectancy [1]. Moreover, it has been indicated that the COVID-19 pandemic has significantly increased the risk of global new-onset DM in both children and adults [25]. AI assisted DR screening allows real-time classification of severity of DR at the point-of-care, in any clinical setting, irrespective of availability of skilled ophthalmic personnel; this in turn allows patients to be informed of any need for onward referral sooner than can be achieved with human graders. AI based DR screening systems accelerate the analysis of fundus images and provides DR grading results at faster than human grader-based systems [31]. AI based DR screening systems can be effectively utilized to improve the efficiency of a screening program and relieves the pressure on eye health systems with regard to training and retention of skilled human graders [32].

Early detection and timely treatment are of paramount importance in DR care pathway. Sight loss due to DR can have profound effects on quality of life of a person with diabetes [33]. Efficient DR screening strategies are needed to reduce patient waiting time, as well as to facilitate more regular follow up. Moreover, AI-driven DR screening allows for more flexibility in relation to location of DR screening. The maturation of digital technologies for retinal imaging coupled with AI has created an ideal environment to deploy AI based eye disease screening. AI based DR screening systems provide effective solutions to already over-burdened ophthalmic or diabetes clinical settings, and advanced the use in non-ophthalmic settings [34, 35]. As it is likely that human-based grading workflows may delay between screening and assessment of results, AI screening provides instant results. Moreover A1 systems have been shown to have 87% sensitivity and 91% specificity [34], providing rapid results, has the potential of offering immediate patient counselling and eye health education in needed, is devoid of human error and has the potential to reduce workload and improve efficiency, can be employed in poorer settings and increasing adherence to follow-up care and reducing the risk of preventable blindness [36, 37]. Additionally, real-world deployment of autonomous AI in large integrated healthcare systems suggest improvements in overall DR screening adherence, patient access, and health equity [9]. A cluster randomised trial conducted in Bangladesh shows that autonomous artificial intelligence yields 40% higher productivity in diabetic eye examinations compared to physician examination. In this trial 100% of the participants were satisfied with the time-to-receive results in AI arm [38]. Although considerable attention has been given to interventions aimed at improving attendance at DR screening [39], less research has focused on strategies to increase the uptake of follow-up appointments after the initial screening is completed. Our meta-analysis demonstrates that AI assisted DR screening improves the uptake of follow-up DR appointments compared to the conventional method of screening using human graders.

AI driven DR screening allows real-time provision of results of the retinal examination at the point-of-care of screening service delivery. Hence it can be integrated in diabetes clinics and non-communicable disease clinics to facilitates patient centred comprehensive care. This approach aligns with World Health Organization recommendation of providing integrated, people-centred eye care [40]. There is substantial potential for using AI to improve DR care pathways, particularly in LMICs where there is a limited sufficient level of skilled human resources for manual grading of retinal images. To enable this to be implemented, more local contextual framework is needed to implement such an AI based screening program if an existing digital surveillance systems for DR does not exist [41]. AI based DR screening strategies might reduce inequalities in access to DR screening [42] and facilitate universal eye health coverage.

A study conducted in Singapore based on the Singapore Integrated Diabetic Retinopathy Programme (SiDRP), a national level programme, showed that semi-automated models using deep learning systems are cost-effective and could save $15 per patient compared with the conventional screening models using human graders [43]. However, the cost-effectiveness of DR screening using AI may depend on country income setting and AI deployment strategy. While current evidence base is more in favour of AI as a cost-effective strategy for DR screening [44–46], its implementation requires readiness of digital retinal imaging systems which require high capital investment [47] and acceptance by the service providers and service users. A meta-analysis reported that AI algorithms can achieve high level of accuracy i.e., sensitivity of 95.3% and specificity of 92.1% for classifying referrable level DR, in real world settings [47]. Reinforcing the current evidence base in favour of deploying AI for DR screening, our review findings show that AI improves uptake of onward referral appointments.

One major limitation of the studies included in this review is lack of data on ungradable images, hence we do not know the rate of dropouts in these DR screening programmes. A scoping review conducted to assess the diagnostic test accuracy and usability of AI in LMICs reported that nearly 31 studies had excluded ungradable images when reporting the outcomes of AI in DR screening [48]. Gradeability of images are important in achieving a high population coverage in DR screening using digital imaging strategies, especially in LMICs where there is high prevalence of cataract [49]. Cataract and corneal diseases in LMICs are major barriers to acquiring effective coverage of digital surveillance using AI. In addition, barriers such as lack of electricity, lack of internet facilities and poor transportation systems hinders wide accessibility for digital screening. Another limitation in our study is lack of data on diagnostic accuracy of each AI or human grader model. Our meta-analysis provides the summary estimates of uptake of DR screening only and does not provide data on level of accuracy of the grading findings. From a DR screening programme implementation point of view, diagnostic test accuracy of each AI model is important to correctly classify different grades of DR. In addition, there is scarce data of AI systems for grading the different severity levels of diabetic macular oedema. The primary studies included our systematic review are also limited by the lack of adherence to reporting guidelines of utilizing AI in research studies such as CONSORT-AI (Consolidated Standard of Reporting Trials - Artificial Intelligence extension) [50]. Studies, especially RCTs using AI in ophthalmology should adhere to the standard reporting guidelines that will improve generalizability of the results. There was only one study conducted in an LMIC (i.e., Rwanda) which limits the generalizability of our findings to resource poor LMIC settings where AI is likely to have significant potential owing to limited skilled human resources. Therefore, there is a need to test these AI based strategies in more diverse test cohorts and national / regional settings before policy planning and implementation [51]. The two studies describing DR screening uptake among youth are from the same setting, but conducted the studies in two different time frames, using different study designs.

## Conclusions

The present systematic review and meta-analysis suggest that DR screening using AI is associated with higher uptake of follow-up examination for referred DR in youth and adults. This is most likely due to instant results being made available with AI based algorithms when compared to a delay in the communication assessment outcomes achieved with human graders.

## Summary

### What was known before

- Diabetic retinopathy (DR) is a common cause of vision loss in diabetic patients, and early detection is crucial to prevent blindness.
- Adherence to follow-up screenings after initial DR detection remains low.
- AI-assisted DR screening offers a cost-effective and efficient alternative to traditional human grading, with similar accuracy.
- The effect of AI-based screening on follow-up adherence has not been thoroughly studied.

### What this study adds

- This study highlights the positive impact of AI-assisted diabetic retinopathy screening on improving patient adherence to follow-up appointments.
- It demonstrates that AI-based screening is more effective than traditional methods in ensuring timely patient follow-up.
- The findings suggest that integrating AI into screening programs could reduce the incidence of vision loss associated with diabetic retinopathy.

## Supplementary information

SUPPLEMENTAL MATERIAL

## Data Availability

The data that support the findings of this study are available from the corresponding author upon reasonable request.
